# Comprehensive review of ICD-9 code accuracies to measure multimorbidity in administrative data

**DOI:** 10.1186/s12913-020-05207-4

**Published:** 2020-06-01

**Authors:** Melissa Y. Wei, Jamie E. Luster, Chiao-Li Chan, Lillian Min

**Affiliations:** 1 Division of General Medicine, Department of Internal Medicine, University of Michigan, 2800 Plymouth Road, Bldg 16, Rm 430W, Ann Arbor, MI 48109 USA; 2 Institute for Healthcare Policy and Innovation, University of Michigan, Ann Arbor, MI USA; 3 Division of Geriatric and Palliative Medicine, Department of Internal Medicine, University of Michigan, Ann Arbor, MI USA; 4 VA Ann Arbor Healthcare System and the Geriatric Research Education Clinical Center (GRECC), Ann Arbor, MI USA

**Keywords:** Multimorbidity, ICD-9, Validation, Literature review

## Abstract

**Background:**

Quantifying the burden of multimorbidity for healthcare research using administrative data has been constrained. Existing measures incompletely capture chronic conditions of relevance and are narrowly focused on risk-adjustment for mortality, healthcare cost or utilization. Moreover, the measures have not undergone a rigorous review for how accurately the components, specifically the International Classification of Diseases, Ninth Revision (ICD-9) codes, represent the chronic conditions that comprise the measures. We performed a comprehensive, structured literature review of research studies on the accuracy of ICD-9 codes validated using external sources across an inventory of 81 chronic conditions. The conditions as a weighted measure set have previously been demonstrated to impact not only mortality but also physical and mental health-related quality of life.

**Methods:**

For each of 81 conditions we performed a structured literature search with the goal to identify 1) studies that externally validate ICD-9 codes mapped to each chronic condition against an external source of data, and 2) the accuracy of ICD-9 codes reported in the identified validation studies. The primary measure of accuracy was the positive predictive value (PPV). We also reported negative predictive value (NPV), sensitivity, specificity, and kappa statistics when available. We searched PubMed and Google Scholar for studies published before June 2019.

**Results:**

We identified studies with validation statistics of ICD-9 codes for 51 (64%) of 81 conditions. Most of the studies (47/51 or 92%) used medical chart review as the external reference standard. Of the validated using medical chart review, the median (range) of mean PPVs was 85% (39–100%) and NPVs was 91% (41–100%). Most conditions had at least one validation study reporting PPV ≥70%.

**Conclusions:**

To help facilitate the use of patient-centered measures of multimorbidity in administrative data, this review provides the accuracy of ICD-9 codes for chronic conditions that impact a universally valued patient-centered outcome: health-related quality of life. These findings will assist health services studies that measure chronic disease burden and risk-adjust for comorbidity and multimorbidity using patient-centered outcomes in administrative data.

## Background

Health system tools for quantifying the burden of multimorbidity (multiple coexisting chronic conditions) have frequently been limited to mortality-based measures such as the Charlson-Deyo comorbidity index and Elixhauser comorbidity score [1–4]. These measures select from a narrow inventory of conditions based on inpatient diagnoses that may not be suitable for capturing the full breadth and depth of multimorbidity across the total healthcare system, including community-dwelling adults receiving ambulatory care. Further, these measures have not undergone a rigorous review for how accurately their components, specifically the International Classification of Diseases, Ninth Revision (ICD-9) codes, represent the clinical presence or absence of a chronic condition.

Modern administrative data-based measures of multimorbidity are needed, along with a firm understanding of the accuracy of the ICD-9 code components used in measures. Among the most comprehensive of multimorbidity measures is an index consisting of 81 chronic conditions assessed longitudinally with repeated measures of self-reported chronic conditions among community-dwelling adults [5]. This multimorbidity-weighted index was developed from three large cohorts of community-dwelling adults with repeated measures of highly reliable self-reported physician-diagnosed chronic conditions and physical health-related quality of life (HRQOL). The index was externally validated in the nationally-representative Health and Retirement Study, with additional physical and cognitive functioning outcomes assessed to demonstrate construct validity [6]. Finally, the conditions were mapped to ICD-9 codes and validated in HRS-Medicare data to facilitate use in claims data [7]. These 81 conditions also predict mortality, HRQOL, social participation, and disability [6, 8–12]. Compared with existing indices, this measure captures a wider breadth of chronic conditions that are prevalent among patients with multimorbidity and spans the widest distribution of multimorbidity at both the low and high extremes of disease burden [6, 8–11].

We aimed to perform a comprehensive array of literature searches to quantify the accuracy of ICD-9 codes used to identify the presence or absence of chronic conditions that impact HRQOL for use in measuring multimorbidity. For each condition, we reviewed the literature to identify: 1) studies that externally validate ICD-9 codes for each condition against an external source of clinical data such as patient interview or medical chart review, and 2) the range of accuracies reported in these validation studies.

## Methods

### Candidate condition selection

We examined 81 chronic conditions in the multimorbidity-weighted index that predicts HRQOL [5] and created a comprehensive mapping of ICD-9 codes for each of these conditions [7] (Additional File, Table 1).

### Study selection

**Fig. 1:**
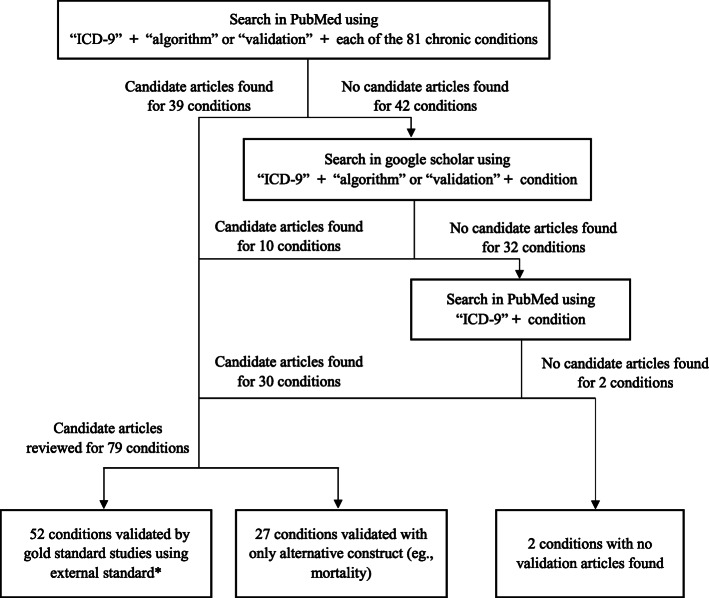
Structured Literature Review Flow Diagram. *We prioritize the articles with the following: 1) chart abstraction as gold standard; 2) self-report, disease registry, or disease screening as gold standard; 3) systematic review. We excluded articles with an algorithm including other criteria than just ICD-9 codes, such as ICD-10 codes, ICD-8 codes, Current Procedural Technology (CPT) codes, lab results, or medications, and those validating ICD-9 codes for multiple conditions in a comorbidity index (e.g., Charlson comorbidity index)

Our primary goal was to conduct a literature search to identify studies that provided validation statistics, i.e., positive predictive value (PPV), negative predictive value (NPV), sensitivity, specificity, or kappa, for ICD-9 codes corresponding to chronic conditions that impact HRQOL. We used PubMed as the primary database, using search terms “ICD-9” and “algorithm” or “validation” combined with each condition (e.g., “ICD-9” AND “atrial fibrillation” AND “validation” OR “algorithm”) through June 2019 (Fig. 1). If no articles were found in PubMed, we searched Google Scholar using the same search terms.

For remaining conditions whose searches failed to identify validation studies, our last tier search (using only “ICD-9” and the condition in PubMed) was to identify studies that used algorithms of ICD-9 codes to identify the chronic condition in administrative data. If the studies used the codes to find associations between the condition with clinical outcomes, we considered that study as evidence supporting construct validity for that ICD-9 algorithm. We reviewed article titles and abstracts to determine if ICD-9 codes for each respective condition might be included in the article, and if applicable, reviewed the full text of the article.

### Inclusion criteria

We had two inclusion criteria for included studies. The first was for the validation of ICD-9 codes (test of interest), and the second was for the reference standard used for the validation (gold standard of interest) (Additional file, Table 2).

### Test of interest

Although some conditions can be further confirmed using other types of administrative billing data (e.g, Current Procedural Technology codes or pharmacy fill data), these sources of data beyond ICD-9 diagnostic codes are not relevant for all conditions and therefore beyond the scope of these review. Therefore, if all algorithms in a study required information beyond ICD-9 codes, we excluded that study.

### Gold standard of interest

We identified studies that used medical chart review (i.e., physician review of nursing, physician, and consultation notes; admission and discharge reports; laboratory and diagnostic test reports; surgical reports; and other clinical and administrative documentation) as the external reference standard because it is the most thorough assessment of several sources of information to confirm the diagnosis of a chronic condition and its associated ICD-9 code in a person. If validation with chart review was unavailable for a given condition, we used those validated by other standards. In order of priority, the secondary reference standards were: 1) self-report, 2) disease registry, and 3) disease screening. For a few conditions we found systematic reviews of validation studies. To include these systematic reviews, we incorporated the aggregate results reported by the study, such as means and ranges.

### Validation statistics

**Fig. 2:**
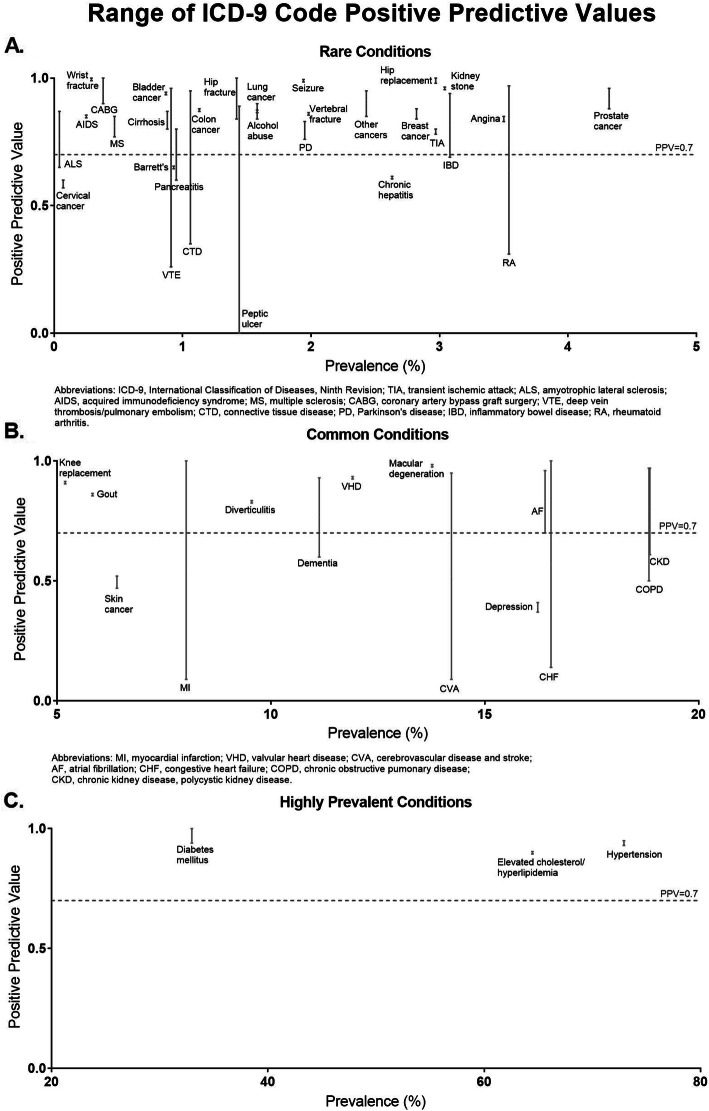
Range of ICD-9 Code Positive Predictive Values

**Fig. 3:**
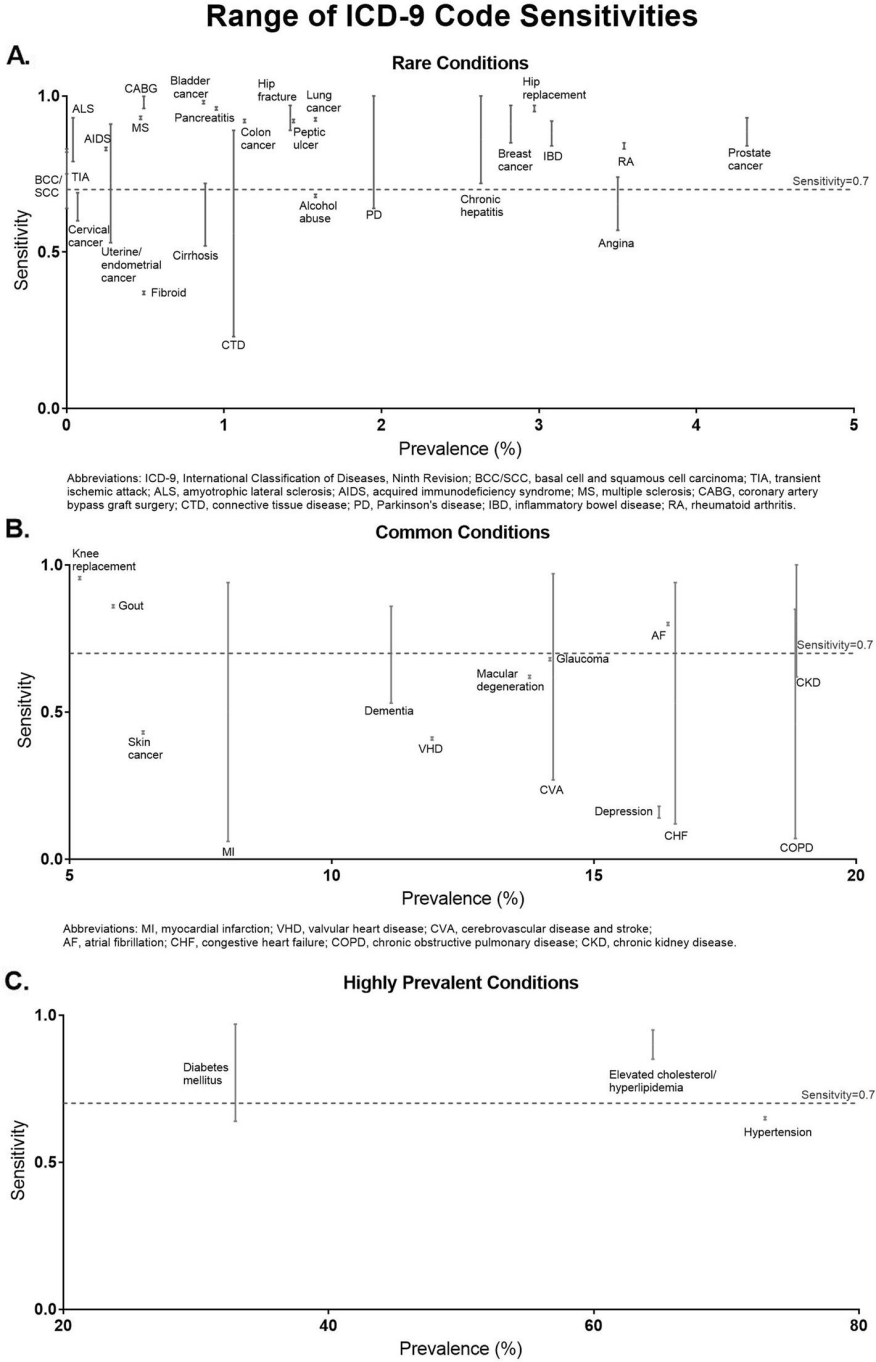
Range of ICD-9 Code Sensitivities

We reported the median and range of the PPV, NPV, sensitivity, specificity, and kappa for each condition for validation studies that provided these metrics. Condition-specific medians were computed by calculating the median of each respective validation statistic of all studies found for a specific condition. The range was computed by reporting the minimum and maximum values of each respective validation statistic of all studies found for a specific condition. The PPV was computed as the probability of being a case (true disease using medical chart review) among those who had a positive screening test (based on ICD-9 codes of interest). The NPV was computed as the probability of not being a case among those with a negative screening test. We plotted the range of PPVs and sensitivities for each condition in three separate graph panels: rare conditions (< 5% prevalence), common conditions (5–20% prevalence), and highly prevalent conditions (> 20% prevalence) (Figs. 2, 3). We used 70% to denote a moderately accurate PPV or sensitivity, for the purposes of displaying the PPVs in Fig. 2 [13, 14] and the sensitivities in Fig. 3.

Prevalence was determined using any ICD-9 codes available for each condition [7] from 8933 Health and Retirement Study participants who provided access to their Medicare outpatient, inpatient, or skilled nursing facility claims in 2014. ICD-9 codes for the 10 most prevalent conditions are summarized in Table 1.

**Table 1:** Multimorbidity-Weighted Index Conditions and the Accuracy, Source, and Type of Validation for Respective ICD-9 Codes for the 10 Most Prevalent Conditions in Medicare claims; 2014

Organ system	Diagnosis	ICD-9 codes found in literature search	Source of Validation^**a**^	Record Type	Sample Size in Study	Algorithm	Accuracy (Sensitivity, Specificity, PPV, NPV, Kappa^**b**^)
Cardiovascular	Hypertension	401.x-405.xx	Chart review	Disease registry	184	2 claims	SENS: 65% SPEC: 90% PPV: 95% NPV: 50% Kappa: 0.45 [15]
		401.x-405.xx, 437.2; exclusion: 250.xx, 430–436, 437.1, 437.9, 438.xx	Chart review	Inpatient, outpatient	76	Any claim up to 9 claims	PPV: 93% [16]
Endocrine	Elevated cholesterol/Hyperlipidemia	272.0–272.4	Chart review	Outpatient	1176	≥1 outpatient	SENS: 85% SPEC: 85% Kappa: 0.70 [17]
		272.0–272.4	Chart review	Disease registry	184	2 claims	SENS: 95% SPEC: 90% PPV: 90% NPV: 95% Kappa: 0.85 [15]
Ophthalmologic	Cataract	366–366.4	Construct validity [18]	N/A			
		13.11, 13.19, 13.20, 13.30, 13.41, 13.42, 13.43, 13.59, 13.69, 13.71	Construct validity [19]	N/A			
Endocrine	Diabetes	250.xx	Chart review	Outpatient	1176	≥1 outpatient claim	SENS: 97% SPEC: 96% Kappa: 0.92 [17]
		250.xx	Chart review	Disease registry	184	2 claims	SENS: 91% SPEC: 100% PPV: 100% NPV: 90% Kappa: 0.90 [15]
		250.00–250.93	Chart review	Any encounter	465	(1) One claim; (2) Two claims	SENS: (1) 92%; (2) 64% SPEC: (1) 99%; (2) 99% PPV: (1) 94%; (2) 95% [20]
Musculoskeletal	Osteoarthrosis	715.xx	Construct validity [21]	N/A			
Endocrine	Hypothyroidism	244.0, 244.1, 244.9	Construct validity [22]	N/A			
Renal	Chronic kidney disease, polycystic kidney disease	403.xx, 405.× 1, 582.xx, 583.xx, 585.x, 586, 593.9	Chart review	Outpatient	1176	≥1 outpatient claim	SENS: 62% SPEC: 98% Kappa: 0.62 [17]
		585.x, 586 (chronic renal failure)	Chart review	Inpatient	7050	(1) Any claim; (2) Primary claim	SENS: (1) 83%; (2) 85% PPV: (1) 70%; (2) 61% [23]
		585.x, 403.xx, 404.xx, 583.81, 581.81, 250.40, 250.42, 250.80, 250.82	Chart review	Inpatient, outpatient	1186	Any claim	PPV: 63% NPV: 54% [24]
		753.12, 753.13, 753.14 (Polycystic kidney disease)	Chart review	Disease registry	184	2 claims	SENS: 100% SPEC: 90% PPV: 90% NPV: 100% Kappa: 0.90 [15]
		(1) 250.40, 250.41, 250.42, 250.43 (diabetic nephropathy); (2) 403.xx, 404.xx (hypertensive nephropathy); (3) 572.4, 580.xx, 584.xx, 580.0, 580.4, 580.89, 580.9, 582.4, 791.2, 791.3 (acute renal failure); (4) 582.xx, 583.xx, 585.x, 586, 587 (chronic renal insufficiency)	Chart review	Medicare claims	1852	Any claim	SEN: (1) 3%; (2) 7%; (3) 5%; (4) 12% SPEC: (1) 99%; (2) 99; (3) 100%; (4) 99% PPV: (1) 86%; (2) 95%; (3) 97%; (4) 97% NPV: (1) 32%; (2) 33%; (3) 33%; (4) 35% [25]
Pulmonary	Chronic obstructive pulmonary disease (COPD)	491.xx-494.x, 496	Chart review	Inpatient	7050	(1) Any claim; (2) Primary claim	SENS: (1) 85%; (2) 79% PPV: (1) 87%; (2) 68% [23]
		491.xx, 492.x, 496	Chart review	Inpatient, outpatient	12,127	(1) ≥2 outpatient or ≥ 1 inpatient; (2) ≥3 outpatient or ≥ 2 inpatient	PPV: (1) 64%; (2) 72% [26]
		491.xx, 492.x, 496	Chart review	Inpatient	1221	Primary claim of 491, 492, or 496	PPV: 50% [17]
		490–492.x, 493.22, 496, 518.81, 518.82, 518.84, 799.1	Chart review	Inpatient	(1) 50; (2) 46; (3) 29; (4) 20	(1) Age ≥ 25, Primary claim of 490, 491.x, 492.x, 493.22, 496OR Primary claim of 518.81, 518.82, 518.84, 799.1 AND secondary claim of 490, 491.x, 492.x, 493.22, 496; (2) Age ≥ 40, Primary claim of 491.x (except for 491.20), 492.x, 493.22, 496 OR primary claim of 518.81, 518.82, 518.84 AND secondary claim of 491.x (except for 491.20), 492.x, 493.22, 496; (3) Age ≥ 40, primary claim of 491.x, 492.x, 496; (4) Age ≥ 40, primary claim of 491.21	SENS: (1) 25%; (2) 24%; (3) 15%; (4) 12% SPEC: (1) 100%; (2) 100%; (3) 100%; (4) 100% PPV: (1) 81%; (2) 85%; (3) 86%; (4) 97% NPV: (1) 94%; (2) 94%; (3) 93%; (4) 93% [27]
		490, 491.xx (all except 491.8), 492.x, 493.xx, 496	Chart review	Outpatient	1176	≥1 outpatient claim	SENS: 81% SPEC: 92% Kappa: 0.68 [28]
Cardiovascular	Congestive heart failure	(1) 398.91, 402.×1, 404.× 1, 404.× 3, or 428.xx; (2) 428.xx	Chart review	Inpatient	908	(1) Any claim; (2) Primary claim	SENS: (1A) 94%; (1B) 55%; (2A) 91%; (2B) 48% PPV: (1A) 43%; (1B) 86%; (2A) 43%; (2B) 86% [29]
		398.91, 402.01, 402.11, 402.91, 404.01, 404.03, 404.11, 404.13, 404.91, 404.93, 425.x, 428.xx	Chart review	Inpatient	497	Primary claim	SENS: 20% SPEC: 100% PPV: 79% NPV: 94% [30]
		398.91, 402.01, 402.11, 402.91, 404.01, 404.03, 404.11, 404.13, 404.91, 404.93, 425.4–425.9, 428.xx	Chart review	Inpatient, outpatient	172	(1) Any claim in 12-month window; (2) Any claim in 24-month window	SENS: (1) 90%; (2) 90% SPEC: (1) 95%; (2) 94% PPV: (1) 72%; (2) 69% NPV: (1) 99%; (2) 98% [31]
		(1) 428.xx; (2) 402.xx or 428.xx; (3) 398.97, 402.×1, 404.xx, 415.0, 416.9, 425.4, 428.xx, 429.4, 514, 518.4, 786.0x	Chart review	Inpatient	5083	Any claim	SENS: (1) 63%; (2) 66%; (3) 67% SPEC: (1) 95%; (2) 93%; (3) 93% PPV: (1) 84%; (2) 79%; (3) 77% NPV: (1) 87%; (2) 88%; (3) 88% [32]
		398.91, 402.×1, 402.×3, 404.× 1, 404.×3, 422.90, 425.4, 425.9, 428.xx	Chart review	Inpatient, outpatient	400	≥1 Primary Discharge or ≥ 3 Secondary Discharge or ≥ 2 Outpatient or ≥ 3 ED or ≥ 2 Secondary Discharge + ≥1 Outpatient Claim	SENS: 62% SPEC: 99% PPV: 69% NPV: 98% [33]
		398.91, 402.01, 402.11, 402.91, 404.01, 404.03, 404.11, 404.13, 404.91, 404.93, 414.8, 428.xx	Chart review	Outpatient	1176	≥1 outpatient	SENS: 77% SPEC: 99% Kappa: 0.74 [17]
		428.xx	Chart review	Disease registry	184	2 claims	SENS: 87% SPEC: 100% PPV: 100% NPV: 85% Kappa: 0.85 [15]
		402.01, 402.11, 402.91, 428.0–428.9x	Chart review	Inpatient	7050	(1) Any claim; (2) Primary claim	SENS: (1) 89%; (2) 85% PPV: (1) 71%; (2) 87% [23]
		(1) 428.xx; (2) 428.xx with other codes; (3) 402.01, 402.11, 425.x, 429.3, and 514	Systematic review	Inpatient, outpatient	35	Various algorithms due to systematic review	PPV: (1) 84–100%; (2) 77–79%; (3) 14–30% [34]
Cardiovascular	Atrial fibrillation	427.31 (atrial fibrillation) or 427.32 (atrial flutter)	Chart review	Inpatient, outpatient, emergency department	300	1 inpatient or 2 outpatient or emergency department	PPV: 96% [35]
		427.3x	Chart review	Outpatient	1176	≥1 outpatient	SENS: 80% SPEC: 99% Kappa: 0.81 [17]
		427.31, 427.32	Systematic review	Inpatient, outpatient	16	Various algorithms due to systematic review	SENS: 57–95% PPV: 70–96% [34]

^a^If the source of validation was a systematic review; the sample size refers to the number of studies included

^b^The number within the bracket following the accuracy values indicates the citation number for the reference

## Results

We considered 81 conditions mapped to ICD-9 codes (Additional File, Table 1). We combined two conditions (basal cell and squamous cell carcinoma) into one group during the literature review once we found no publications validating codes for these conditions separately. This resulted in a final validation for 80 total conditions. After mapping all conditions to corresponding ICD-9 codes, we found articles providing validation statistics for codes for 51 (64%) of the 80 conditions. We also found articles with validating through construct validity for 27 (34%) of the 80 conditions. We did not find articles validating codes for 2 (2.5%) of the 80 conditions.

Medical chart review was the most common method of ICD-9 code validation (47 of 51 conditions with validation statistics, 92%), followed by systematic review (5 conditions, 10%), self-report of condition (1 condition, 2%), disease registry (1 condition, 2%), and diagnostic screening (1 condition, 2%), not mutually exclusive.

Of the 51 conditions reporting validation statistics, the median and range for the accuracies were as follows: sensitivity 83% (3–100%, *n* = 142 values), specificity 97% (0–100%, *n* = 76), PPV 84% (0–100%, *n* = 175), NPV 90% (32–100%, *n* = 52), and kappa statistic 0.85 (0.45–0.92, *n* = 18) (Additional file, Table 1). The most common validation measure reported was PPV (available for 46 conditions), followed by sensitivity (available for 43 conditions).

Most ICD-9 coded conditions had moderate to high mean PPV and NPVs of at least 70% (37/46, 80%) among studies that provided PPVs, and 19/24 (79%) among studies that provided NPVs). We observed variation in the reported accuracies, with the highest mean PPV for wrist fracture (100%) compared with the lowest mean PPV for depression (39%). The highest mean NPV was for chronic hepatitis/hepatocellular disease (100%), and the lowest was for Parkinson disease (41%).

We plotted the ranges of PPVs and sensitivities for chronic conditions mapped to ICD codes from least to greatest disease prevalence (Figs. 2, 3). Of the 46 conditions that provided PPV as a validation metric, 42 (91%) had at least one publication reporting a PPV ≥70%. Myocardial infarction had the widest range of PPVs (9–100%). Of the 44 conditions that provided sensitivity, 33 (75%) had at least one publication reporting a sensitivity of ≥70%. Myocardial infarction had the widest range of sensitivities (6–94%).

For conditions that provided codes validated through construct validity, we present the respective ICD-9 codes based on mapping these conditions from ICD code mappings with CMS fiscal year 2015 (October 1, 2014 to September 30, 2015) ICD-9-CM, a comprehensive list of ICD-9 codes and corresponding conditions. Codes were identified and checked independently for agreement by four individuals (including authors MYW, JEL, CC). To validate the accuracy of these mapped conditions without validation studies and the overall mapping of ICD-9 codes to conditions in the multimorbidity-weighted index, we examined the construct validity and conducted direct comparisons of the ICD-coded multimorbidity-weighted index with traditional metrics [7].

## Discussion

This literature review provides evidence to support the accuracy and validity of using ICD-9 diagnostic codes to classify the presence or absence of 81 chronic conditions to measure multimorbidity in administrative-based data. The ICD-9 codes we studied had overall moderate to high PPVs and NPVs (≥70%) based on external standards for presence versus absence of each individual condition. This may be attributed to several factors such as different population samples, coding artifact [36, 37], and different methodologic approaches and comprehensiveness with mapping ICD-9 codes to conditions.

The highest priority reference standard, medical chart review, was available for 47 of 81 conditions.

This study provides researchers with a tool to code many existing indices, as well as one using all 81 conditions that has previously been demonstrated to predict HRQOL [10]. We also feature an innovative approach to the challenging task of synthesizing the results from 81 separate literature reviews by presenting results graphically by prevalence, for added context and face validity. To our knowledge, a comprehensive review for this scope of chronic conditions has not been performed previously. In addition, prior reviews [38] focus on largely inpatient conditions that predict mortality, but this review captures chronic conditions prevalent among community-dwelling adults with multimorbidity that impact physical and mental HRQOL.

Administrative and claims-based studies using ICD-9 codes have traditionally relied upon a few commonly used measures for comorbidity adjustment that weight conditions to mortality risk, healthcare cost and utilization. Measures such as the Charlson-Deyo and Elixhauser comorbidity measures [1–3] have been readily available in datasets, facilitating and perpetuating their use. However, a possible unintended consequence of convenience has been overextending their intended application such as risk-adjustment for other outcomes such as HRQOL.

### Strengths and limitations

Although existing comorbidity indices are available for use in administrative data and have frequently been extrapolated to measure multimorbidity, this research provides a practical method to operationalize a modern measure of multimorbidity that predicts a universal health outcome, physical functioning. Previous comorbidity indices have been limited in the scope of included conditions and are calibrated to outcomes of limited relevance to disease survivors, such as inpatient mortality risk, healthcare cost and utilization [2–4, 39, 40]. This inventory of 81 chronic conditions is one of the most comprehensive multimorbidity indices and is a validated, patient-centered measure of multimorbidity that assigns disease severity based on the impact conditions have on physical functioning, an outcome of particular relevance for older adults. Additional strengths over existing indices include a broad distribution, greater precision in quantifying multimorbidity, and rigorous validation for predicting several downstream consequences of multimorbidity [6–10]. As patient-centered health outcomes persist in importance and relevance for research and policy, this review offers an additional tool to measure and target outcomes for quality improvement. For example, the ICD-9 codes presented in this study could be used as a proxy for physical functioning, which is absent in administrative data.

Our study has limitations. First, we focused only on chronic condition diagnostic codes to define test variables. However, the accuracy of some conditions such as diabetes might be further improved by adding laboratory values available in an electronic health record. Other administrative data, such as procedure codes and durable medical equipment, could also potentially augment the ICD codes, but were beyond the scope of this review. Second, our literature search did not include articles published in languages other than English or articles not accessible through PubMed or Google Scholar. Google Scholar can access 87% of all scholarly documents online, including journal and conference papers, dissertations, books, technical reports, and working papers and identified more documents than PubMed, Web of Science, and Microsoft Academic Search [41]. Third, these validated ICD-9 codes apply to retrospective quality improvement efforts prior to 2016 using existing data available through the Centers for Medicare and Medicaid Services. Application to future prospective research requires a cross-walk of the conditions to ICD-10 codes and validation, which are underway. Finally, the overall value of any measure of multimorbidity in administrative data will depend on the completeness and accuracy of documentation by providers, which is a limitation inherent to all claims data. For example, financial incentives have been suspected as the cause of biased coding practices over time [37].

### Implications for further research

Our findings demonstrate that there is variation in the quality and accuracy of ICD code mappings for chronic conditions, including some conditions that lack external validity. Future studies are needed to validate the accuracy of ICD codes for conditions that did not provide these measures of accuracy design, analysis, interpretation of results, and applications to clinical care and health policy. To increase the accuracy of diagnoses with ICD codes, one future direction for researchers would be to include data beyond ICD codes such as medications, labs, and imaging studies when consistently available for specific chronic conditions.

## Conclusion

Modern measures of multimorbidity that weight disease severity by patient-centered outcomes have emerged and are available for use in administrative studies. We provide a comprehensive inventory of diagnostic codes mapped to chronic conditions that impact HRQOL. This research demonstrates moderate to high accuracies and validation for most but not all diagnostic codes. Based on our comprehensive literature reviews, researchers can apply with a fuller understanding of the validity of diagnostic codes for specific chronic diseases to identify populations with multimorbidity or risk-adjust for comorbidity using a comprehensive and patient-centered measure.

## Supplementary information

**Additional file 1 : Additional File Table 1.** Multimorbidity-Weighted Index Conditions and the Accuracy, Source, and Type of Validation for Respective ICD-9 Codes. **Additional File Table 2.** Two by Two Table for the Association Between ICD Codes (Test) to Indicate a Chronic Condition and an External Reference Standard (Gold Standard) to Verify a Chronic Condition.

## Data Availability

The datasets analyzed during the current study are available in the Health and Retirement Study repository, http://hrsonline.isr.umich.edu.

## Abbreviations

HRQOL: Health-related quality of life
ICD-9: International Classification of Diseases, Ninth Revision
NPV: Negative predictive value
PPV: Positive predictive value
N/A: Not available
SEN: Sensitivity
SPEC: Specificity
ICD-10: International Classification of Diseases, Tenth Revision
ICD-8: International Classification of Diseases, Eighth Revision
CPT: Current procedural technology
AIDS: Acquired immunodeficiency syndrome
ALS: Amyotrophic lateral sclerosis
CABG: Coronary artery bypass graft surgery
CTD: Connective tissue disease
IBD: Inflammatory bowel disease
MS: Multiple sclerosis
PD: Parkinson disease
RA: Rheumatoid arthritis
TIA: Transient ischemic attack
VTE: Venous thromboembolism (deep vein thrombosis/pulmonary embolism)
AF: Atrial fibrillation
CHF: Congestive heart failure
CKD: Chronic kidney disease
COPD: Chronic obstructive pulmonary disease
CVA: Cerebrovascular disease and stroke
MI: Myocardial infarction
VHD: Valvular heart disease
CC/SCC: Basal cell carcinoma and squamous cell carcinoma

## References

[1] Deyo RA, Cherkin DC, Ciol MA (1992). Adapting a clinical comorbidity index for use with ICD-9-CM administrative databases. J Clin Epidemiol.

[2] Charlson ME, Pompei P, Ales KL, MacKenzie CR (1987). A new method of classifying prognostic comorbidity in longitudinal studies: development and validation. J Chronic Dis.

[3] Elixhauser A, Steiner C, Harris DR, Coffey RM (1998). Comorbidity measures for use with administrative data. Med Care.

[4] Romano PS, Roos LL, Jollis JG (1993). Adapting a clinical comorbidity index for use with ICD-9-CM administrative data: differing perspectives. J Clin Epidemiol.

[5] Wei MY, Kawachi I, Okereke OI, Mukamal KJ (2016). Diverse cumulative impact of chronic diseases on physical health-related quality of life: implications for a measure of multimorbidity. Am J Epidemiol.

[6] Wei MY, Kabeto MU, Langa KM, Mukamal KJ (2018). Multimorbidity and physical and cognitive function: performance of a new multimorbidity-weighted index. J Gerontol A Biol Sci Med Sci.

[7] Wei MY, Ratz D, Mukamal KJ. Multimorbidity in Medicare Beneficiaries: Performance of an ICD-Coded Multimorbidity-Weighted Index. J Am Geriatr Soc. 2020. 10.1111/jgs.16310 [Epub ahead of print]. 10.1111/jgs.16310 PMC7234913 31917465

[8] Wei MY, Mukamal KJ (2018). Multimorbidity, mortality, and long-term physical functioning in 3 prospective cohorts of community-dwelling adults. Am J Epidemiol.

[9] Wei MY, Kabeto MU, Galecki AT, Langa KM (2019). Physical functioning decline and mortality in older adults with multimorbidity: joint modeling of longitudinal and survival data. J Gerontol A Biol Sci Med Sci.

[10] Wei MY, Mukamal KJ (2019). Multimorbidity and mental health-related quality of life and risk of completed suicide. J Am Geriatr Soc.

[11] Wei MY, Levine DA, Zahodne LB, Kabeto MU, Langa KM. Multimorbidity and cognitive decline over 14 years in older Americans. J Gerontol A Biol Sci Med Sci. 2019. 10.1093/gerona/glz147. 10.1093/gerona/glz147 PMC7243582 31173065

[12] Luster J, Ratz D, Wei M. Predictors of social participation among middle-aged and older Americans: data from the health and retirement study. Philadelphia: American public health association annual meeting; 2019.

[13] Jensen PN, Johnson K, Floyd J, Heckbert SR, Carnahan R, Dublin S (2012). A systematic review of validated methods for identifying atrial fibrillation using administrative data. Pharmacoepidemiol Drug Saf.

[14] Thigpen JL, Dillon C, Forster KB, Henault L, Quinn EK, Tripodis Y (2015). Validity of international classification of disease codes to identify ischemic stroke and intracranial hemorrhage among individuals with associated diagnosis of atrial fibrillation. Circ Cardiovasc Qual Outcomes.

[15] Navaneethan SD, Jolly SE, Schold JD, Arrigain S, Saupe W, Sharp J (2011). Development and validation of an electronic health record-based chronic kidney disease registry. Clin J Am Soc Nephrol.

[16] Tamariz L, Palacio A, Denizard J, Schulman Y, Contreras G (2015). The use of claims data algorithms to recruit eligible participants into clinical trials. Am J Manag Care.

[17] Lacasse Y, Daigle JM, Martin S, Maltais F (2012). Validity of chronic obstructive pulmonary disease diagnoses in a large administrative database. Can Respir J.

[18] Muir KW, Gupta C, Gill P, Stein JD (2013). Accuracy of international classification of diseases, ninth revision, clinical modification billing codes for common ophthalmic conditions. JAMA Ophthalmol.

[19] Gray DT, Hodge DO, Ilstrup DM, Butterfield LC, Baratz KH (1997). Concordance of Medicare data and population-based clinical data on cataract surgery utilization in Olmsted County. Minnesota Am J Epidemiol.

[20] Wilson C, Susan L, Lynch A, Saria R, Peterson D (2001). Patients with diagnosed diabetes mellitus can be accurately identified in an Indian Health Service patient registration database. Public Health Rep.

[21] Cisternas MG, Murphy L, Sacks JJ, Solomon DH, Pasta DJ, Helmick CG (2016). Alternative methods for defining osteoarthritis and the impact on estimating prevalence in a US population-based survey. Arthritis Care Res.

[22] Lin HJ, Lin CC, Lin HM, Chen HJ, Lin CC, Chang CT (2018). Hypothyroidism is associated with all-cause mortality in a national cohort of chronic haemodialysis patients. Nephrology (Carlton).

[23] Fisher ES, Whaley FS, Krushat WM, Malenka DJ, Fleming C, Baron JA (1992). The accuracy of Medicare's hospital claims data: progress has been made, but problems remain. Am J Public Health.

[24] Nadkarni GN, Gottesman O, Linneman JG, Chase H, Berg RL, Farouk S (2014). Development and validation of an electronic phenotyping algorithm for chronic kidney disease. AMIA Annu Symp Proc.

[25] Winkelmayer WC, Schneeweiss S, Mogun H, Patrick AR, Avorn J, Solomon DH. Identification of individuals with CKD from Medicare claims data: a validation study. Am J Kidney Dis. 2005;46(2):225–32. 10.1053/j.ajkd.2005.04.029. 10.1053/j.ajkd.2005.04.029 16112040

[26] Ho TW, Ruan SY, Huang CT, Tsai YJ, Lai F, Yu CJ (2018). Validity of ICD9-CM codes to diagnose chronic obstructive pulmonary disease from National Health Insurance claim data in Taiwan. Int J Chron Obstruct Pulmon Dis.

[27] Stein BD, Bautista A, Schumock GT, Lee TA, Charbeneau JT, Lauderdale DS (2012). The validity of international classification of diseases, ninth revision, clinical modification diagnosis codes for identifying patients hospitalized for COPD exacerbations. Chest..

[28] Borzecki AM, Wong AT, Hickey EC, Ash AS, Berlowitz DR (2004). Identifying hypertension-related comorbidities from administrative data: what's the optimal approach?. Am J Med Qual.

[29] Rosenman M, He J, Martin J, Nutakki K, Eckert G, Lane K (2014). Database queries for hospitalizations for acute congestive heart failure: flexible methods and validation based on set theory. J Am Med Inform Assoc.

[30] Presley CA, Min JY, Chipman J, Greevy RA, Grijalva CG, Griffin MR (2018). Validation of an algorithm to identify heart failure hospitalisations in patients with diabetes within the veterans health administration. BMJ Open.

[31] Floyd JS, Blondon M, Moore KP, Boyko EJ, Smith NL (2016). Validation of methods for assessing cardiovascular disease using electronic health data in a cohort of veterans with diabetes. Pharmacoepidemiol Drug Saf.

[32] Goff DC, Pandey DK, Chan FA, Ortiz C, Nichaman MZ (2000). Congestive heart failure in the United States: is there more than meets the I (CD code)? The Corpus Christi heart project. Arch Intern Med.

[33] Allen LA, Yood MU, Wagner EH, Aiello Bowles EJ, Pardee R, Wellman R (2014). Performance of claims-based algorithms for identifying heart failure and cardiomyopathy among patients diagnosed with breast cancer. Med Care.

[34] Saczynski JS, Andrade SE, Harrold LR, Tjia J, Cutrona SL, Dodd KS (2012). A systematic review of validated methods for identifying heart failure using administrative data. Pharmacoepidemiol Drug Saf.

[35] Navar-Boggan AM, Rymer JA, Piccini JP, Shatila W, Ring L, Stafford JA (2015). Accuracy and validation of an automated electronic algorithm to identify patients with atrial fibrillation at risk for stroke. Am Heart J.

[36] Schneeweiss S, Avorn J (2005). A review of uses of health care utilization databases for epidemiologic research on therapeutics. J Clin Epidemiol.

[37] Silva GC, Jiang L, Gutman R, Wu W-C, Mor V, Fine MJ (2020). Mortality trends for veterans hospitalized with heart failure and pneumonia using claims-based vs clinical risk-adjustment variables. JAMA Intern Med.

[38] Quan H, Li B, Couris CM, Fushimi K, Graham P, Hider P (2011). Updating and validating the Charlson comorbidity index and score for risk adjustment in hospital discharge abstracts using data from 6 countries. Am J Epidemiol.

[39] Centers for Medicare and Medicaid Services. CMS Chronic Conditions Data Warehouse (CCW). https://www.ccwdata.org/web/guest/home. Accessed 18 July 2019.

[40] Agency for Healthcare Research and Quality. Chronic Condition Indicator (CCI) for ICD-9-CM. http://www.hcup-us.ahrq.gov/toolssoftware/chronic/chronic.jsp#overview. Accessed 18 July 2019.

[41] Khabsa M, Giles CL. The number of scholarly documents on the public web. PLoS One. 9(5):e93949-e. 10.1371/journal.pone.0093949. 10.1371/journal.pone.0093949 PMC4015892 24817403

