# Supplementary material for: Comprehensive review of ICD-9 code accuracies to measure multimorbidity in administrative data
Source: BMC Health Serv Res. 2020 Jun 1;20:489. doi: 10.1186/s12913-020-05207-4 (PMC7268621; doi:10.1186/s12913-020-05207-4)
Supplement: Supplementary file 1 — Additional file 1 : Additional File Table 1. Multimorbidity-Weighted Index Conditions and the Accuracy, Source, and Type of Validation for Respective ICD-9 Codes. Additional File Table 2. Two by Two Table for the Association Between ICD Codes (Test) to Indicate a Chronic Condition and an External Reference Standard (Gold Standard) to Verify a Chronic Condition. [file 12913_2020_5207_MOESM1_ESM.docx]

Additional File Table 1. Multimorbidity-Weighted Index Conditions and the Accuracy, Source, and Type of Validation for Respective ICD-9 Codes.

| **Organ System** | **Diagnosis** | **ICD-9 codes found in literature search** | **Source of Validation^b^** | **Record Type** | **Sample Size in Study** | **Algorithm** | **Accuracy (Sensitivity, Specificity, PPV, NPV, Kappa^c^)** |  |
| --- | --- | --- | --- | --- | --- | --- | --- | --- |
| Cardiovascular | 1. Atrial fibrillation | 427.31 (atrial fibrillation) or 427.32 (atrial flutter) | Chart review | Inpatient, outpatient, emergency department | 300 | 1 inpatient or 2 outpatient or emergency department | PPV: 96%[1] |  |
|  |  | 427.3x | Chart review | Outpatient | 1176 | ≥1 outpatient | SENS: 80% SPEC: 99% Kappa: 0.81[2] |  |
|  |  | 427.31, 427.32 | Systematic review | Inpatient, outpatient | 16 | Various algorithms due to systematic review | SENS: 57-95%  PPV: 70-96%[3] |  |
|  | 2. Automated Implantable Cardioverter Defibrillator (AICD) | 37.94, 37.96 | Construct validity [4] | N/A | | | |  |
|  | 3. Aneurysm/dissection of chest area | 441.3, 441.4, 441.5, 441.9, 38.44; excluding 441.0x, 441.1, 747.1x, 759.8x, 758.6, 446.0 | Construct validity [5] | N/A | | | |  |
|  | 4. Angina | 413.x (except 413.0) | Chart review | Inpatient, outpatient | 172 | (1) Any claim in 12-month window;  (2) Any claim in 24-month window | SENS: (1) 57%; (2) 74% SPEC: (1) 99%; (2) 99%  PPV: (1) 83%; (2) 85%  NPV: (1) 96%; (2) 97%[6] |  |
|  | 5. Coronary artery bypass graft surgery | 36.1x | Chart review | Inpatient | 7050 | (1) Any claim;  (2) Primary claim | SENS: (1) 96%; (2) 100% PPV: (1) 100%; (2) 90%[7] |  |
|  | 6. Congestive heart failure | (1) 398.91, 402.x1, 404.x1, 404.x3, or 428.xx; (2) 428.xx | Chart review | Inpatient | 908 | (1) Any claim; (2) Primary claim | SENS: (1A) 94%; (1B) 55%; (2A) 91%; (2B) 48% PPV: (1A) 43%; (1B) 86%; (2A) 43%; (2B) 86%[8] |  |
|  |  | 398.91, 402.01, 402.11, 402.91, 404.01, 404.03, 404.11,  404.13, 404.91, 404.93, 425.x, 428.xx | Chart review | Inpatient | 497 | Primary claim | SENS: 20% SPEC: 100% PPV: 79% NPV: 94%[9] |  |
|  |  | 398.91, 402.01, 402.11, 402.91, 404.01, 404.03, 404.11, 404.13, 404.91, 404.93, 425.4-425.9, 428.xx | Chart review | Inpatient, outpatient | 172 | (1) Any claim in 12-month window;  (2) Any claim in 24-month window | SENS: (1) 90%; (2) 90% SPEC: (1) 95%; (2) 94%  PPV: (1) 72%; (2) 69%  NPV: (1) 99%; (2) 98%[6] |  |
|  |  | (1) 428.xx; (2) 402.xx or 428.xx; (3) 398.97, 402.x1, 404.xx, 415.0, 416.9,  425.4, 428.xx, 429.4, 514, 518.4, 786.0x | Chart review | Inpatient | 5083 | Any claim | SENS: (1) 63%; (2) 66%; (3) 67% SPEC: (1) 95%; (2) 93%; (3) 93% PPV: (1) 84%; (2) 79%; (3) 77% NPV: (1) 87%; (2) 88%; (3) 88%[10] |  |
|  |  | 398.91, 402.x1, 402.x3, 404.x1, 404.x3, 422.90, 425.4, 425.9, 428.xx | Chart review | Inpatient, outpatient | 400 | ≥1 Primary Discharge or ≥3 Secondary Discharge or ≥2 Outpatient or ≥3 ED or ≥2 Secondary Discharge + ≥1 Outpatient Claim | SENS: 62% SPEC: 99% PPV: 69% NPV: 98%[11] |  |
|  |  | 398.91, 402.01, 402.11, 402.91, 404.01, 404.03, 404.11, 404.13, 404.91, 404.93, 414.8, 428.xx | Chart review | Outpatient | 1176 | ≥1 outpatient | SENS: 77% SPEC: 99% Kappa: 0.74[2] |  |
|  |  | 428.xx | Chart review | Disease registry | 184 | 2 claims | SENS: 87% SPEC: 100% PPV: 100% NPV: 85% Kappa: 0.85[12] |  |
|  |  | 402.01, 402.11, 402.91, 428.0-428.9x | Chart review | Inpatient | 7050 | (1) Any claim;  (2) Primary claim | SENS: (1) 89%; (2) 85% PPV: (1) 71%; (2) 87%[7] |  |
|  |  | (1) 428.xx; (2) 428.xx with other codes; (3) 402.01, 402.11, 425.x, 429.3, and 514 | Systematic review | Inpatient, outpatient | 35 | Various algorithms due to systematic review | PPV: (1) 84-100%; (2) 77-79%; (3) 14-30%[13] |  |
|  | 7. Hypertension | 401.x-405.xx | Chart review | Disease registry | 184 | 2 claims | SENS: 65% SPEC: 90% PPV: 95% NPV: 50% Kappa: 0.45[12] |  |
|  |  | 401.x-405.xx, 437.2; exclusion: 250.xx, 430-436, 437.1, 437.9, 438.xx | Chart review | Inpatient, outpatient | 76 | Any claim up to 9 claims | PPV: 93%[14] |  |
|  | 8. Myocardial infarction | 410.xx, 412 | Chart review | Inpatient, outpatient | 172 | (1) Any claim in 12-month window;  (2) Any claim in 24-month window | SENS: (1) 38%; (2) 44% SPEC: (1) 100%; (2) 100% PPV: (1) 97%; (2) 98% NPV: (1) 87%; (2) 88%[6] |  |
|  |  | 410.xx, 411.xx | Chart review | Inpatient | 956 | Primary claim of (1) 410; (2) 411 | PPV: (1) 95%; (2) 9%[15] |  |
|  |  | 410.xx | Chart review | Inpatient | 17900 | Primary or secondary claim; (1) male; (2) female | SENS: (1) 65%; (2) 60% PPV: (1) 77%; (2) 73%[16] |  |
|  |  | 410.x0, 410.x1 | Chart review | Inpatient | 143 | Primary claim | PPV: 86%[17] |  |
|  |  | 410.x0, 410.x1 | Chart review | Inpatient | 103 | (1) Primary claim;  (2) Secondary claim;  (3) Primary, secondary, or unspecified claim | PPV: (1) 93%; (2) 88%; (3) 75%[18] |  |
|  |  | 410.xx | Chart review | Inpatient | 154 | Any claim | SENS: 67% SPEC: 100% PPV: 100%[19] |  |
|  |  | 410.xx or 411.xx | Chart review | Inpatient | 5329 | Any claim | SENS: 81% SPEC: 93% PPV: 55% NPV: 98%[20] |  |
|  |  | 410.xx | Chart review | Inpatient | 7050 | (1) Any claim;  (2) Primary claim | SENS: (1) 90%; (2) 94% PPV: (1) 87%; (2) 92%[7] |  |
|  | 9.Cerebrovascular disease and stroke | 433.x1, 434.x1, 436, 437.1, or 437.9 | Chart review | Inpatient | 200 | Any claim | PPV: 87%[21] |  |
|  |  | 433.x1, 434.x1 | Chart review | Emergency department, inpatient | 3915 | Primary claim | SENS: 71% PPV: 90%[22] |  |
|  |  | 433.x1, 434.xx, 436 | Chart review | Inpatient | 131 | Primary claim | PPV: 60%[23] |  |
|  |  | (1) 433.xx-434.xx, 436 (ischemic stroke)  (2) 430-432.x (intracranial hemorrhage) | Chart review | Inpatient | 1812 | Any claim | PPV: (1) 94%; (2) 95%[24] |  |
|  |  | 431, 432.9, 434.xx-434.9x, 436 | Chart review | Inpatient | 7050 | (1) Any claim;  (2) Primary claim | SENS: (1) 92%; (2) 94% PPV: (1) 71%; (2) 81%[7] |  |
|  |  | 430, 431, 433.x1, 434.xx (excluding 434.x0), or 436 | Chart review | Inpatient | 231 | (1) Primary claim; (2) Any claim | PPV: (1) 97%; (2) 89%[25] |  |
|  |  | Tested each ICD-9 code separately: (1) 342.xx; (2) 430; (3) 431; (4) 432.x; (5) 433.xx; (6) 434.xx; (7) 436; (8) 437.x; (9) 438.xx | Chart review | Inpatient | 4015 | Primary or up to 5 secondary claims | PPV: (1) 36%; (2) 76%; (3) 78%; (4) 54%; (5) 9%; (6) 77%; (7) 61%; (8) 14%; (9) 9%[26] |  |
|  | 10. Valvular heart disease | 394.x-397.x, 398.9x, 42.4x, V42.2, V43.3 | Chart review | Inpatient | 10401 | Any claim | SENS: 41% SPEC: 97% PPV: 93% NPV: 68%[27] |  |
| Endocrine | 11. Diabetes | 250.xx | Chart review | Outpatient | 1176 | ≥1 outpatient claim | SENS: 97% SPEC: 96% Kappa: 0.92[2] |  |
|  |  | 250.xx | Chart review | Disease registry | 184 | 2 claims | SENS: 91% SPEC: 100% PPV: 100% NPV: 90% Kappa: 0.90[12] |  |
|  |  | 250.00-250.93 | Chart review | Any encounter | 465 | (1) One claim; (2) Two claims | SENS: (1) 92%; (2) 64% SPEC: (1) 99%; (2) 99% PPV: (1) 94%; (2) 95%[28] |  |
|  | 12. Elevated cholesterol/  Hyperlipidemia | 272.0-272.4 | Chart review | Outpatient | 1176 | ≥1 outpatient | SENS: 85% SPEC: 85% Kappa: 0.70[2] |  |
|  |  | 272.0-272.4 | Chart review | Disease registry | 184 | 2 claims | SENS: 95% SPEC: 90% PPV: 90% NPV: 95% Kappa: 0.85[12] |  |
|  | 13. Hypertriglyceridemia | 272.3 | Construct validity [29] | N/A | | | |  |
|  | 14. Hyperthyroidism | 242.9x | Construct validity [30] | N/A | | | |  |
|  | 15. Hypothyroidism | 244.0, 244.1, 244.9 | Construct validity [31] | N/A | | | |  |
|  | 16. Thyroid nodule/Goiter | 242.0x (diffuse goiter); 241.x, 242.1x, 242.3x (nodular goiter); 241.1, 242.2x (multinodular goiter); 240.x, 246.1 (unspecified goiter); 246.2 (thyroid cyst) | Construct validity [32] | N/A | | | |  |
| Gastrointestinal | 17. Barrett’s esophagus | 530.2, 530.85 | Chart review | Outpatient | 1672 | Any claim | PPV: 65%[33] |  |
|  | 18. Cirrhosis, liver transplant | (1) 571.2, (2) 571.5 | Chart review | Inpatient, outpatient | 2893 | Any claim | SENS: (1) 52%; (2) 72% SPEC: (1) 84%; (2) 68% PPV: (1) 87%; (2) 80% NPV: (1) 46%; (2) 53%[34] |  |
|  | 19. Diverticulosis/ diverticulitis | 562.11, 562.13 | Chart review | Inpatient, outpatient, emergency department | 2117 | Any claim | PPV: 83%[35] |  |
|  | 20. Gallstones |  | N/A for gallstones | | | | |  |
|  | 21. Chronic hepatitis/hepatocellular disease | 070.2x, 070.3x | Chart review | Any encounter | 1,652,055 | Other auxiliary data^a^ | SENS: 84%  SPEC: 100%  PPV: 61%  NPV: 100%[36] |  |
|  | 22. Inflammatory bowel disease | (1) 555.x; (2) 556.x | Chart review | Inpatient, outpatient | 511 | Any claim | SENS: (1) 92%; (2) 84% SPEC: (1) 99%; (2) 99% PPV: (1) 88%; (2) 82%[37] |  |
|  |  | 555.x or 556.x | Chart review | Inpatient, outpatient | 1871 | (1) 1 claim; (2) 5 codes; (3) 2 claims, ≥1 outpatient; (4) 2 outpatient or 1 inpatient; (5) 2 outpatient and 1 inpatient | PPV: (1) 69%; (2) 94%; (3) 91%; (4) 87%; (5) 92%[38] |  |
|  | 23. Pancreatitis | 577.0, excluding 577.1 | Chart review | Inpatient | 802 | Primary claim | SENS: 96% SPEC: 85% PPV: 80% NPV: 98%[39] |  |
|  |  | 577.0 | Systematic review | Inpatient, outpatient | 8 | Various algorithms due to systematic review | PPV: 60-80%  NPV: >90%[40] |  |
|  | 24. Colon polyp |  | N/A for colon polyp | | | | |  |
|  | 25. Ulcer, peptic | (1) 531.xx-534.xx, 578.x; (2) 532.xx; (3) 531.xx, 534.xx; (4) 533.xx | Chart review | Inpatient | 347 | Primary claim | PPV: (1) 46%; (2) 77%; (3) 76%; (4) 0%[41] |  |
|  |  | 531.xx-534.xx | Chart review | Inpatient | 7050 | (1) Any claim;  (2) Primary claim | SENS: (1) 92%; (2) 92% PPV: (1) 75%; (2) 89%[7] |  |
| Hematologic | 26. Pernicious anemia | 281.0 | Construct validity [42] | N/A | | | |  |
| Immunologic | 27. AIDS | 042 (AIDS); V08 (asymptomatic HIV); 042.0-.2, 042.9, 043.0-.3, 043.9, 044.9, 079.53 (HIV-related codes). | Chart review | Any encounter | 1153 | Other auxiliary data^a^ | SENS: 83%  SPEC: 86%  PPV: 85%  NPV: 90%[43] |  |
| Integumentary | 28. Solar actinic keratosis | 702.0 | Construct validity [44] | N/A | | | |  |
| Musculoskeletal | 29. Connective tissue disease | 710.3 | Chart review | Inpatient, outpatient | (1) Outpatient 206; (2) Inpatient primary 21; (3) Inpatient secondary 50 | (1) Outpatient; (2) Inpatient primary claim; (3) Inpatient secondary claim | SENS: (1) 89%; (2) 23%; (3) 26% PPV: (1) 35%; (2) 95%; (3) 46%[45] |  |
|  |  | 710.0 | Chart review | Inpatient, outpatient | 200 | (1) ≥1 claim; (2) ≥2 claims; (3) ≥3 claims; (4) ≥4 claims | SENS: (1) NA; (2) 86%; (3) 77%; (4) 71% PPV: (1) 49%; (2) 65%; (3) 75%; (4) 79%[46] |  |
|  | 30. Herniated disc | 722.10, 722.2 | Construct validity [47] | N/A | | | |  |
|  | 31. Hip fracture | 820.xx | Chart review | Inpatient | 104 | Any claim | PPV: 100%[48] |  |
|  |  | 820.xx | Chart review | Inpatient | 1415 | Primary or secondary claim | SENS: 89% PPV: 84%[49] |  |
|  |  | 820.xx-829.x | Chart review | Inpatient | 7050 | (1) Any claim;  (2) Primary claim | SENS: (1) 96%; (2) 97% PPV: (1) 94%; (2) 96%[7] |  |
|  | 32. Vertebral fracture | 805.xx (spine fracture) | Chart review | Inpatient | 101 | Any claim | PPV: 86%[48] |  |
|  | 33. Wrist fracture | 813.xx-814.xx (forearm/wrist fracture) | Chart review | Inpatient | 94 | Any claim | PPV: 100%[48] |  |
|  | 34. Gout | 274.xx | Chart review | Inpatient, outpatient emergency department/urgent care | 85 | Any claim | SENS: 86% SPEC: 95% PPV: 86% NPV: 95% Kappa: 0.86[50] |  |
|  | 35. Hip replacement surgery | 81.5x, 81.6x | Chart review | Inpatient | 7050 | (1) Any claim;  (2) Primary claim | SENS: (1) 97%; (2) 95% PPV: (1) 100%; (2) 98%[7] |  |
|  | 36. Knee replacement surgery | 00.80-00.84, 81.54-81.55 | Chart review | Any encounter | 200 | Other auxiliary data^a^ | SENS: 96% SPEC: 92% PPV: 91% NPV: 97% Kappa:0.88[51] |  |
|  | 37. Osteoarthrosis | 715.xx | Construct validity [52] | N/A | | | |  |
|  | 38. Osteoporosis | 733.0x (osteoporosis); 805.2, 805.4, 805.8, 733.13, 737.10 (vertebral fractures). | Construct validity [53] | N/A | | | |  |
|  | 39. Rheumatoid arthritis | 714.0-714.33 | Chart review | Inpatient, outpatient | 60 | ≥1 claim | PPV: 97%[54] |  |
|  |  | 714.xx | Chart review | Inpatient, outpatient | (1) ≥2 claims 131; (2) ≥3 claims 110 | (1) ≥2 claims (2) ≥3 claims | PPV: (1) 56%; (2) 66% [55] |  |
|  |  | 714.xx | Chart review | Outpatient | 543 | ≥2 claims at least 6 months apart | PPV: 31%[56] |  |
|  |  | 714.0 | Chart review | Inpatient | 7050 | (1) Any claim;  (2) Primary claim | SENS: (1) 83%; (2) 85% PPV: (1) 81%; (2) 85%[7] |  |
|  |  | 714.xx | Systematic review | Inpatient, outpatient | 9 | Various algorithms due to systematic review | PPV: 34-97%[57] |  |
| Nervous | 40. Amyotrophic lateral sclerosis (ALS) | 335.20 | Chart review | Inpatient | 527 | Primary or secondary claim | SENS: 93% SPEC: 99% PPV: 87% NPV: 99%[58] |  |
|  |  | 335.2x | Chart review | Inpatient | 1,012,122 | Primary claim | SENS: 92% SPEC: 100% PPV: 65%[59] |  |
|  | 41. Dementia including Alzheimer's | 290.0-290.9, 331.0-331.2 | Chart review | Inpatient | 7050 | (1) Any claim;  (2) Primary claim | SENS: (1) 76%; (2) 53% PPV: (1) 60%; (2) 75%[7] |  |
|  |  | 290.0, 290.1x, 290.3, 290.4x, 294.1x, 331.0, 331.1x, 331.82 | Chart review | Inpatient | 340 | Primary or secondary claim | PPV: 93%[60] |  |
|  | 42. Migraine | 346.xx | Construct validity [61-63] | N/A | | | |  |
|  |  |  |  |  |  |  |  |  |
|  |  |  |  |  |  |  |  |  |
|  | 43. Multiple sclerosis | 340 | Chart review | Inpatient, outpatient | 25,712 | ≥1 claim | SENS: 93% SPEC: 92% Kappa: 0.85[64] |  |
|  |  | 377.3x, 323.82, 323, 341.9, 340, 341.0 | Chart review and self-report | Inpatient, outpatient | 400 | (1) ≥7 medical contacts for MS; (2)  ≥3 medical contacts for MS | SENS: (1, self-report) 92%; (2, self-report) 90%  SPEC: (1, self-report) 77%; (2, self-report) 56%  PPV: (1, chart review) 85%; (2, chart review) 77%; (1, self-report) 92%; (2, self-report) 75%  NPV: (1, chart review) 74%; (2, chart review) 76%; (1, self-report) 79%; (2, self-report) 80%  Kappa: (2, self-report) 0.86[65] |  |
|  | 44. Parkinson disease | 332.0 | Chart review | Outpatient | 577 | (1) ≥1 claim; (2) ≥2 claims; (3) ≥5 claims | SENS: (1) 100%; (2) 89%; (3) 67% SPEC: (1) 0%; (2) 28%; (3) 52% PPV: (1) 76%; (2) 79%; (3) 83% NPV: (1) NA; (2) 46%; (3) 36%[66] |  |
|  | 45. Restless legs syndrome | 333.90, 333.99 | Construct validity [67] | N/A | | | |  |
|  | 46. Seizure disorder and epilepsy | 345.xx | Chart review | Inpatient, emergency department, outpatient | 940 | Primary claim | PPV: 99% NPV: 97%[68] |  |
|  | 47. Transient ischemic attack | 435.x | Chart review | Inpatient | (1) Primary claim 130; (2) Any claim 145 | (1) Primary claim; (2) Any claim | SENS: (1) 75%; (2) 82% PPV: (1) 80%; (2) 78%[69] |  |
| Oncologic | 48. Bladder cancer | 88.xx, 233.7, 236.7, 239.4 | Chart review | Inpatient, outpatient | 100 | ≥2 outpatient claims | SENS: 98% SPEC: 90% PPV: 94%[70] |  |
|  | 49. Breast cancer | 174.x | Chart review | Inpatient | 7050 | (1) Any claim;  (2) Primary claim | SENS: (1) 97%; (2) 96% PPV: (1) 84%; (2) 88%[7] |  |
|  | 50. Cervical cancer | 795.04, 622.12, 233.1, 180.1, V73.81 | Chart review | Outpatient | 1713 | (1) ≥1 claim; (2) ≥2 claims | PPV: (1) 57%, (2) 60%[71] |  |
|  | 51. Colon cancer | 153.x-154.x | Chart review | Inpatient | 7050 | (1) Any claim;  (2) Primary claim | SENS: (1) 92%; (2) 92% PPV: (1) 88%; (2) 87%[7] |  |
|  | 52. Lung cancer | 162.x | Chart review | Inpatient | 7050 | (1) Any claim;  (2) Primary claim | SENS: (1) 93%; (2) 92% PPV: (1) 90%; (2) 84%[7] |  |
|  | 53. Leukemia, lymphoma | 200.00-208.91 | Construct validity [72] | N/A | | | |  |
|  | 54. Melanoma | 172.x | Construct validity [73] | N/A | | | |  |
|  | 55. Other cancer | 155.0 (hepatocellular carcinoma) | Chart review | Inpatient, outpatient | 616 | (1) Any claim (2) Any inpatient claim regardless of outpatient claim | SENS: (1) 100%; (2) 72% SPEC: (1) 99%; (2) 100% PPV: (1) 85%; (2) 95% NPV: (1) 100%; (2) 99%[74] |  |
|  | 56. Ovarian cancer | 183.xx | Construct validity [75] | N/A | | | |  |
|  | 57. Prostate cancer | 185 | Chart review | Inpatient | 7050 | (1) Any claim;  (2) Primary claim | SENS: (1) 84%; (2) 93% PPV: (1) 88%; (2) 96%[7] |  |
|  | 58. Basal and squamous cell carcinoma | 173.xx | Chart review | Outpatient | 965 | Any claim | PPV: 47%[76] |  |
|  |  | 173.xx | Chart review | Outpatient | 5995 | Any claim | SENS: 64% SPEC: 85% PPV: 52% NPV: 90%[77] |  |
|  | 59. Uterine/endometrial cancer | 182.0-182.8, 68.8 | Disease registry | Inpatient | 16853 | (1) Primary claim; (2) Any claim | SENS: (1) 77%, (2) 84%[78] |  |
| Ophthalmologic | 60. Cataract | 366-366.4 | Construct validity [79] | N/A | | | |  |
|  |  | 13.11, 13.19, 13.20, 13.30, 13.41, 13.42, 13.43, 13.59, 13.69, 13.71 | Construct validity [80] | N/A | | | |  |
|  | 61. Glaucoma | 365.1x-365.9x, 365.0x | Self-report | Inpatient, outpatient, ancillary | 4613 | Any claim | SENS: 68% SPEC: 95%[81] |  |
|  | 62. Macular degeneration | 362.52 | Chart review | Outpatient | 363 | Any claim | SENS: 62% SPEC: 100% PPV: 98%[82] |  |
| Oral | 63. Gingival and periodontal disease | 523.4x (chronic periodontitis) | Construct validity [83] | N/A | | | |  |
|  |  | 523.1x (chronic gingivitis) | Construct validity [84] | N/A | | | |  |
| Psychiatric | 64. Alcohol abuse | 291.x, 303.xx, 305.0x | Chart review | Inpatient and outpatient | 730 | Any claim | SENS: 68% SPEC: 97% PPV: 87% NPV: 91%[85] |  |
|  | 65. Depression | 296.20–296.24, 296.30–296.34 | Disease screening | Medicare Part A and Part B | 321 | Any claim | SENS: (1) 14%; (2) 18% SPEC: (1) 96%; (2) 93% PPV: (1) 41%; (2) 37% NPV: (1) 84%; (2) 84%[86] |  |
| Pulmonary | 66. Asthma | 493.0x-493.9x | Chart review | Inpatient, outpatient, emergency department | 1,099 | Any claim | SENS: 43% SPEC: 97%[87] |  |
|  | 67. Chronic obstructive pulmonary disease (COPD) | 491.xx-494.x, 496 | Chart review | Inpatient | 7050 | (1) Any claim;  (2) Primary claim | SENS: (1) 85%; (2) 79% PPV: (1) 87%; (2) 68%[7] |  |
|  |  | 491.xx, 492.x, 496 | Chart review | Inpatient, outpatient | 12,127 | (1) ≥2 outpatient or ≥1 inpatient; (2) ≥3 outpatient or ≥2 inpatient | PPV: (1) 64%; (2) 72%[88] |  |
|  |  | 491.xx, 492.x, 496 | Chart review | Inpatient | 1221 | Primary claim of 491, 492, or 496 | PPV: 50%[89] |  |
|  |  | 490-492.x, 493.22, 496, 518.81, 518.82, 518.84, 799.1 | Chart review | Inpatient | 1. 50; (2) 46; (3) 29; (4) 20 | 1. Age≥ 25, Primary claim of 490, 491.x, 492.x, 493.22, 496 OR Primary claim of 518.81, 518.82, 518.84, 799.1 AND secondary claim of 490, 491.x, 492.x, 493.22, 496; (2) Age ≥40, Primary claim of 491.x (except for 491.20), 492.x, 493.22, 496 OR primary claim of 518.81, 518.82, 518.84 AND secondary claim of 491.x (except for 491.20), 492.x, 493.22, 496; (3) Age ≥40, primary claim of 491.x, 492.x, 496; (4) Age ≥40, primary claim of 491.21 | SENS: (1) 25%; (2) 24%; (3) 15%; (4) 12% SPEC: (1) 100%; (2) 100%; (3) 100%; (4) 100% PPV: (1) 81%; (2) 85%; (3) 86%; (4) 97% NPV: (1) 94%; (2) 94%; (3) 93%; (4) 93%[90] |  |
|  |  | 490, 491.xx (all except 491.8), 492.x, 493.xx, 496 | Chart review | Outpatient | 1176 | ≥1 outpatient claim | SENS: 81% SPEC: 92% Kappa: 0.68[2] |  |
|  | 68. Deep vein thrombosis/pulmonary embolism | 415.1x, 451.xx-453.xx, 671.3x, 671.4x, 671.9x, 673.2x, 673.83, 996.73, 996.74, 997.2, 999.2, 996.7x | Systematic review | Inpatient, outpatient | 7 | Various algorithms due to systematic review | PPV: 26%-96%[91] |  |
| Renal | 69. Calculus of kidney and ureter, Calculus of lower urinary tract | 592.0, 592.1, 592.9 | Chart review | Inpatient, outpatient | 98 | Primary, secondary, or third position in claim | PPV: 96%[92] |  |
|  | 70. Chronic kidney disease, polycystic kidney disease | 403.xx, 405.x1, 582.xx, 583.xx, 585.x, 586, 593.9 | Chart review | Outpatient | 1176 | ≥1 outpatient claim | SENS: 62% SPEC: 98% Kappa: 0.62[2] |  |
|  |  | 585.x, 586 (chronic renal failure) | Chart review | Inpatient | 7050 | (1) Any claim;  (2) Primary claim | SENS: (1) 83%; (2) 85% PPV: (1) 70%; (2) 61%[7] |  |
|  |  | 585.x,  403.xx, 404.xx, 583.81, 581.81, 250.40, 250.42, 250.80, 250.82 | Chart review | Inpatient, outpatient | 1186 | Any claim | PPV: 63% NPV: 54%[93] |  |
|  |  | 753.12, 753.13, 753.14 (Polycystic kidney disease) | Chart review | Disease registry | 184 | 2 claims | SENS: 100% SPEC: 90% PPV: 90% NPV: 100% Kappa: 0.90[12] |  |
|  |  | (1) 250.40, 250.41, 250.42, 250.43 (diabetic nephropathy); (2) 403.xx, 404.xx (hypertensive nephropathy); (3) 572.4, 580.xx, 584.xx, 580.0, 580.4, 580.89, 580.9, 582.4, 791.2, 791.3 (acute renal failure); (4) 582.xx, 583.xx, 585.x, 586, 587 (chronic renal insufficiency) | Chart review | Medicare claims | 1852 | Any claim | SEN: (1) 3%; (2) 7%; (3) 5%; (4) 12%  SPEC: (1) 99%; (2) 99; (3) 100%; (4) 99%  PPV: (1) 86%; (2) 95%; (3) 97%; (4) 97%  NPV: (1) 32%; (2) 33%; (3) 33%; (4) 35%[94] |  |
|  | 71. Interstitial and other cystitis | 098.0, 098.2, 595.0, 595.2, 595.9, 599.0, 597.xx (UTI), 590.xx (upper UTI) | Construct validity [95] | N/A | | | |  |
|  |  | 595.1, 595.2, 595.5 | Construct validity [96] | N/A | | | |  |
| Reproductive | 72. Benign prostatic hyperplasia (BPH) | 600.xx | Construct validity [97] | N/A | | | |  |
|  |  | 600.xx | Construct validity [98] | N/A | | | |  |
|  |  | 599.6x, 600.0x, 60.21, 60.29, 60.3, 60.4, 60.94, 60.95, 788.20, 788.21, 788.29, 788.41, 788.42, 788.43, 788.61, 788.62 | Construct validity [99] | N/A | | | |  |
|  | 73. Prostate surgery for BPH | 60.21, 60.29, 60.3, 60.4, 60.94, 60.95 | Construct validity [99] | N/A | | | |  |
|  | 74. Benign breast disease, Disorders of breast | 217, 611.xx | Construct validity [100] | N/A | | | |  |
|  | 75. Erectile dysfunction | 607.84 | Construct validity [83] | N/A | | | |  |
|  | 76. Endometriosis | 617.x | Construct validity [101] | N/A | | | |  |
|  | 77. Uterine fibroid | 218.x, 654.1x | Chart review | Inpatient | 65 | Primary or secondary position in claim | SENS: 37% SPEC: 100%[102] |  |
|  | 78. Polycystic ovary syndrome, Noninflammatory disorders of ovary, fallopian tube, and broad ligament | 706.0, 706.1, 704.0x, 704.1, 626.x, 256.4 | Construct validity [103] | N/A | | | |  |
|  | 79. Premenstrual syndrome | 625.4 | Construct validity [104] | N/A | | | |  |
|  | 80. Ectopic pregnancy | 633.xx | Construct validity [105] | N/A | | | |  |
|  |  |  |  |  |  |  |  |  |

Abbreviations: N/A, not available; NPV, negative predictive value; PPV, positive predictive value; SENS, sensitivity; SPEC, specificity

^a^ Algorithm for case finding included data other than ICD-9 codes alone, but these were the only validation articles found for these conditions so they were included.

^b^ If the source of validation was a systematic review, the sample size refers to the number of studies included.

^c^The number within the bracket following the accuracy values indicates the citation number for the reference.

Additional File Table 2. Two by Two Table for the Association Between ICD Codes (Test) to Indicate a Chronic Condition and an External Reference Standard (Gold Standard) to Verify a Chronic Condition.

|  | Condition indicated by external reference standard | Condition not indicated by external reference standard |
| --- | --- | --- |
| Condition indicated by  ICD codes | True positives | False positives |
| Condition not indicated by ICD codes | False negatives | True negatives |

Abbreviations: ICD-9, International Classification of Diseases, Ninth Revision

^a^Positive predictive value = True positives / (True positives + False positives)

^b^Negative predictive value = True negatives / (True negatives + False negatives)

REFERENCES

1. Navar-Boggan AM, Rymer JA, Piccini JP, Shatila W, Ring L, Stafford JA et al. Accuracy and validation of an automated electronic algorithm to identify patients with atrial fibrillation at risk for stroke. American heart journal. 2015;169(1):39-44 e2.

2. Borzecki AM, Wong AT, Hickey EC, Ash AS, Berlowitz DR. Identifying hypertension-related comorbidities from administrative data: what's the optimal approach? American journal of medical quality : the official journal of the American College of Medical Quality. 2004;19(5):201-6.

3. Jensen PN, Johnson K, Floyd J, Heckbert SR, Carnahan R, Dublin S. A systematic review of validated methods for identifying atrial fibrillation using administrative data. Pharmacoepidemiol Drug Saf. 2012;21 Suppl 1:141-7.

4. Bradshaw PJ, Stobie P, Briffa T, Hobbs MS. Use and long-term outcomes of implantable cardioverter-defibrillators, 1990 to 2009. American heart journal. 2013;165(5):816-22.

5. Katz DJ, Stanley JC, Zelenock GB. Operative mortality rates for intact and ruptured abdominal aortic aneurysms in Michigan: an eleven-year statewide experience. Journal of vascular surgery. 1994;19(5):804-15; discussion 816-7.

6. Floyd JS, Blondon M, Moore KP, Boyko EJ, Smith NL. Validation of methods for assessing cardiovascular disease using electronic health data in a cohort of Veterans with diabetes. Pharmacoepidemiology and drug safety. 2016;25(4):467-71.

7. Fisher ES, Whaley FS, Krushat WM, Malenka DJ, Fleming C, Baron JA et al. The accuracy of Medicare's hospital claims data: progress has been made, but problems remain. American journal of public health. 1992;82(2):243-8.

8. Rosenman M, He J, Martin J, Nutakki K, Eckert G, Lane K et al. Database queries for hospitalizations for acute congestive heart failure: flexible methods and validation based on set theory. Journal of the American Medical Informatics Association : JAMIA. 2014;21(2):345-52.

9. Presley CA, Min JY, Chipman J, Greevy RA, Grijalva CG, Griffin MR et al. Validation of an algorithm to identify heart failure hospitalisations in patients with diabetes within the veterans health administration. BMJ open. 2018;8(3):e020455.

10. Goff DC, Jr., Pandey DK, Chan FA, Ortiz C, Nichaman MZ. Congestive heart failure in the United States: is there more than meets the I(CD code)? The Corpus Christi Heart Project. Archives of internal medicine. 2000;160(2):197-202.

11. Allen LA, Yood MU, Wagner EH, Aiello Bowles EJ, Pardee R, Wellman R et al. Performance of claims-based algorithms for identifying heart failure and cardiomyopathy among patients diagnosed with breast cancer. Medical care. 2014;52(5):e30-8.

12. Navaneethan SD, Jolly SE, Schold JD, Arrigain S, Saupe W, Sharp J et al. Development and validation of an electronic health record-based chronic kidney disease registry. Clinical journal of the American Society of Nephrology : CJASN. 2011;6(1):40-9.

13. Saczynski JS, Andrade SE, Harrold LR, Tjia J, Cutrona SL, Dodd KS et al. A systematic review of validated methods for identifying heart failure using administrative data. Pharmacoepidemiol Drug Saf. 2012;21 Suppl 1:129-40.

14. Tamariz L, Palacio A, Denizard J, Schulman Y, Contreras G. The use of claims data algorithms to recruit eligible participants into clinical trials. The American journal of managed care. 2015;21(2):e114-8.

15. Varas-Lorenzo C, Castellsague J, Stang MR, Tomas L, Aguado J, Perez-Gutthann S. Positive predictive value of ICD-9 codes 410 and 411 in the identification of cases of acute coronary syndromes in the Saskatchewan Hospital automated database. Pharmacoepidemiology and drug safety. 2008;17(8):842-52.

16. Rosamond WD, Chambless LE, Sorlie PD, Bell EM, Weitzman S, Smith JC et al. Trends in the sensitivity, positive predictive value, false-positive rate, and comparability ratio of hospital discharge diagnosis codes for acute myocardial infarction in four US communities, 1987-2000. American journal of epidemiology. 2004;160(12):1137-46.

17. Cutrona SL, Toh S, Iyer A, Foy S, Daniel GW, Nair VP et al. Validation of acute myocardial infarction in the Food and Drug Administration's Mini-Sentinel program. Pharmacoepidemiology and drug safety. 2013;22(1):40-54.

18. Ammann EM, Schweizer ML, Robinson JG, Eschol JO, Kafa R, Girotra S et al. Chart validation of inpatient ICD-9-CM administrative diagnosis codes for acute myocardial infarction (AMI) among intravenous immune globulin (IGIV) users in the Sentinel Distributed Database. Pharmacoepidemiology and drug safety. 2018;27(4):398-404.

19. McAlpine R, Pringle S, Pringle T, Lorimer R, MacDonald TM. A study to determine the sensitivity and specificity of hospital discharge diagnosis data used in the MICA study. Pharmacoepidemiology and drug safety. 1998;7(5):311-8.

20. Pladevall M, Goff DC, Nichaman MZ, Chan F, Ramsey D, Ortiz C et al. An assessment of the validity of ICD Code 410 to identify hospital admissions for myocardial infarction: The Corpus Christi Heart Project. International journal of epidemiology. 1996;25(5):948-52.

21. Wahl PM, Rodgers K, Schneeweiss S, Gage BF, Butler J, Wilmer C et al. Validation of claims-based diagnostic and procedure codes for cardiovascular and gastrointestinal serious adverse events in a commercially-insured population. Pharmacoepidemiology and drug safety. 2010;19(6):596-603.

22. Baldereschi M, Balzi D, Di Fabrizio V, De Vito L, Ricci R, D'Onofrio P et al. Administrative data underestimate acute ischemic stroke events and thrombolysis treatments: Data from a multicenter validation survey in Italy. PloS one. 2018;13(3):e0193776.

23. Ammann EM, Leira EC, Winiecki SK, Nagaraja N, Dandapat S, Carnahan RM et al. Chart validation of inpatient ICD-9-CM administrative diagnosis codes for ischemic stroke among IGIV users in the Sentinel Distributed Database. Medicine. 2017;96(52):e9440.

24. Thigpen JL, Dillon C, Forster KB, Henault L, Quinn EK, Tripodis Y et al. Validity of international classification of disease codes to identify ischemic stroke and intracranial hemorrhage among individuals with associated diagnosis of atrial fibrillation. Circulation Cardiovascular quality and outcomes. 2015;8(1):8-14.

25. Roumie CL, Mitchel E, Gideon PS, Varas-Lorenzo C, Castellsague J, Griffin MR. Validation of ICD-9 codes with a high positive predictive value for incident strokes resulting in hospitalization using Medicaid health data. Pharmacoepidemiology and drug safety. 2008;17(1):20-6.

26. Spolaore P, Brocco S, Fedeli U, Visentin C, Schievano E, Avossa F et al. Measuring accuracy of discharge diagnoses for a region-wide surveillance of hospitalized strokes. Stroke; a journal of cerebral circulation. 2005;36(5):1031-4.

27. Birman-Deych E, Waterman AD, Yan Y, Nilasena DS, Radford MJ, Gage BF. Accuracy of ICD-9-CM codes for identifying cardiovascular and stroke risk factors. Medical care. 2005;43(5):480-5.

28. Wilson C, Susan L, Lynch A, Saria R, Peterson D. Patients with diagnosed diabetes mellitus can be accurately identified in an Indian Health Service patient registration database. Public health reports (Washington, DC : 1974). 2001;116(1):45-50.

29. Gaudet D, Signorovitch J, Swallow E, Fan L, Tremblay K, Brisson D et al. Medical resource use and costs associated with chylomicronemia. Journal of medical economics. 2013;16(5):657-66.

30. Huang SW, Lin JW, Wang WT, Wu CW, Liou TH, Lin HW. Hyperthyroidism is a risk factor for developing adhesive capsulitis of the shoulder: a nationwide longitudinal population-based study. Scientific reports. 2014;4:4183.

31. Lin HJ, Lin CC, Lin HM, Chen HJ, Lin CC, Chang CT et al. Hypothyroidism is associated with all-cause mortality in a national cohort of chronic haemodialysis patients. Nephrology (Carlton, Vic). 2018;23(6):559-564.

32. Huang LY, Lee YL, Chou P, Chiu WY, Chu D. Thyroid fine-needle aspiration biopsy and thyroid cancer diagnosis: a nationwide population-based study. PloS one. 2015;10(5):e0127354.

33. Corley DA, Kubo A, DeBoer J, Rumore GJ. Diagnosing Barrett's esophagus: reliability of clinical and pathologic diagnoses. Gastrointestinal endoscopy. 2009;69(6):1004-10.

34. Nehra MS, Ma Y, Clark C, Amarasingham R, Rockey DC, Singal AG. Use of administrative claims data for identifying patients with cirrhosis. Journal of clinical gastroenterology. 2013;47(5):e50-4.

35. Kawatkar A, Chu LH, Iyer R, Yen L, Chen W, Erder MH et al. Development and validation of algorithms to identify acute diverticulitis. Pharmacoepidemiology and drug safety. 2015;24(1):27-37.

36. Mahajan R, Moorman AC, Liu SJ, Rupp L, Klevens RM. Use of the International Classification of Diseases, 9th revision, coding in identifying chronic hepatitis B virus infection in health system data: implications for national surveillance. Journal of the American Medical Informatics Association : JAMIA. 2013;20(3):441-5.

37. Thirumurthi S, Chowdhury R, Richardson P, Abraham NS. Validation of ICD-9-CM diagnostic codes for inflammatory bowel disease among veterans. Digestive diseases and sciences. 2010;55(9):2592-8.

38. Hou JK, Tan M, Stidham RW, Colozzi J, Adams D, El-Serag H et al. Accuracy of diagnostic codes for identifying patients with ulcerative colitis and Crohn's disease in the Veterans Affairs Health Care System. Digestive diseases and sciences. 2014;59(10):2406-10.

39. Saligram S, Lo D, Saul M, Yadav D. Analyses of hospital administrative data that use diagnosis codes overestimate the cases of acute pancreatitis. Clinical gastroenterology and hepatology : the official clinical practice journal of the American Gastroenterological Association. 2012;10(7):805-811.e1.

40. Moores K, Gilchrist B, Carnahan R, Abrams T. A systematic review of validated methods for identifying pancreatitis using administrative data. Pharmacoepidemiology and drug safety. 2012;21 Suppl 1:194-202.

41. Andrade SE, Gurwitz JH, Chan KA, Donahue JG, Beck A, Boles M et al. Validation of diagnoses of peptic ulcers and bleeding from administrative databases: a multi-health maintenance organization study. Journal of clinical epidemiology. 2002;55(3):310-3.

42. Brinton LA, Gridley G, Hrubec Z, Hoover R, Fraumeni JF, Jr. Cancer risk following pernicious anaemia. British journal of cancer. 1989;59(5):810-3.

43. Goetz MB, Hoang T, Kan VL, Rimland D, Rodriguez-Barradas M. Development and validation of an algorithm to identify patients newly diagnosed with HIV infection from electronic health records. AIDS research and human retroviruses. 2014;30(7):626-33.

44. Neugebauer R, Levandoski KA, Zhu Z, Sokil M, Chren MM, Friedman GD et al. A real-world, community-based cohort study comparing the effectiveness of topical fluorouracil versus topical imiquimod for the treatment of actinic keratosis. Journal of the American Academy of Dermatology. 2018;78(4):710-716.

45. Kwa MC, Ardalan K, Laumann AE, Nardone B, West DP, Silverberg JI. Validation of International Classification of Diseases Codes for the Epidemiologic Study of Dermatomyositis. Arthritis care & research. 2017;69(5):753-757.

46. Barnado A, Casey C, Carroll RJ, Wheless L, Denny JC, Crofford LJ. Developing Electronic Health Record Algorithms That Accurately Identify Patients With Systemic Lupus Erythematosus. Arthritis care & research. 2017;69(5):687-693.

47. Kardaun JW, White LR, Shaffer WO. Acute complications in patients with surgical treatment of lumbar herniated disc. Journal of spinal disorders. 1990;3(1):30-8.

48. Sing CW, Woo YC, Lee ACH, Lam JKY, Chu JKP, Wong ICK et al. Validity of major osteoporotic fracture diagnosis codes in the Clinical Data Analysis and Reporting System in Hong Kong. Pharmacoepidemiology and drug safety. 2017;26(8):973-976.

49. Lofthus CM, Cappelen I, Osnes EK, Falch JA, Kristiansen IS, Medhus AW et al. Local and national electronic databases in Norway demonstrate a varying degree of validity. Journal of clinical epidemiology. 2005;58(3):280-5.

50. Singh JA. Veterans Affairs databases are accurate for gout-related health care utilization: a validation study. Arthritis research & therapy. 2013;15(6):R224.

51. Singh JA, Ayub S. Accuracy of VA databases for diagnoses of knee replacement and hip replacement. Osteoarthritis and cartilage. 2010;18(12):1639-42.

52. Cisternas MG, Murphy L, Sacks JJ, Solomon DH, Pasta DJ, Helmick CG. Alternative Methods for Defining Osteoarthritis and the Impact on Estimating Prevalence in a US Population-Based Survey. Arthritis care & research. 2016;68(5):574-80.

53. Gehlbach SH, Fournier M, Bigelow C. Recognition of osteoporosis by primary care physicians. American journal of public health. 2002;92(2):271-3.

54. Jafri K, Taylor L, Nezamzadeh M, Baker JF, Mehta NN, Bartels C et al. Management of hyperlipidemia among patients with rheumatoid arthritis in the primary care setting. BMC musculoskeletal disorders. 2015;16:237.

55. Kim SY, Servi A, Polinski JM, Mogun H, Weinblatt ME, Katz JN et al. Validation of rheumatoid arthritis diagnoses in health care utilization data. Arthritis research & therapy. 2011;13(1):R32.

56. Ng B, Aslam F, Petersen NJ, Yu HJ, Suarez-Almazor ME. Identification of rheumatoid arthritis patients using an administrative database: a Veterans Affairs study. Arthritis care & research. 2012;64(10):1490-6.

57. Chung CP, Rohan P, Krishnaswami S, McPheeters ML. A systematic review of validated methods for identifying patients with rheumatoid arthritis using administrative or claims data. Vaccine. 2013;31 Suppl 10:K41-61.

58. Pisa FE, Verriello L, Deroma L, Drigo D, Bergonzi P, Gigli GL et al. The accuracy of discharge diagnosis coding for Amyotrophic Lateral Sclerosis in a large teaching hospital. European journal of epidemiology. 2009;24(10):635-40.

59. Beghi E, Logroscino G, Micheli A, Millul A, Perini M, Riva R et al. Validity of hospital discharge diagnoses for the assessment of the prevalence and incidence of amyotrophic lateral sclerosis. Amyotrophic lateral sclerosis and other motor neuron disorders : official publication of the World Federation of Neurology, Research Group on Motor Neuron Diseases. 2001;2(2):99-104.

60. van de Vorst IE, Vaartjes I, Sinnecker LF, Beks LJ, Bots ML, Koek HL. The validity of national hospital discharge register data on dementia: a comparative analysis using clinical data from a university medical centre. The Netherlands journal of medicine. 2015;73(2):69-75.

61. Altalib HH, Fenton BT, Sico J, Goulet JL, Bathulapalli H, Mohammad A et al. Increase in migraine diagnoses and guideline-concordant treatment in veterans, 2004-2012. Cephalalgia. 2017;37(1):3-10.

62. Bigal ME, Kolodner KB, Lafata JE, Leotta C, Lipton RB. Patterns of medical diagnosis and treatment of migraine and probable migraine in a health plan. Cephalalgia. 2006;26(1):43-9.

63. Weinreich HM, Carey JP. Prevalence of Pulsatile Tinnitus Among Patients With Migraine. Otol Neurotol. 2016;37(3):244-7.

64. Culpepper WJ, 2nd, Ehrmantraut M, Wallin MT, Flannery K, Bradham DD. Veterans Health Administration multiple sclerosis surveillance registry: The problem of case-finding from administrative databases. Journal of rehabilitation research and development. 2006;43(1):17-24.

65. Marrie RA, Yu N, Blanchard J, Leung S, Elliott L. The rising prevalence and changing age distribution of multiple sclerosis in Manitoba. Neurology. 2010;74(6):465-71.

66. Szumski NR, Cheng EM. Optimizing algorithms to identify Parkinson's disease cases within an administrative database. Movement disorders : official journal of the Movement Disorder Society. 2009;24(1):51-6.

67. Yang FC, Lin TY, Chen HJ, Lee JT, Lin CC, Kao CH. Risk of Restless Legs Syndrome Following Tension-Type Headache: A Nationwide Population-Based Cohort Study. Medicine (Baltimore). 2015;94(46):e2109.

68. Jette N, Reid AY, Quan H, Hill MD, Wiebe S. How accurate is ICD coding for epilepsy? Epilepsia. 2010;51(1):62-9.

69. Leone MA, Capponi A, Varrasi C, Tarletti R, Monaco F. Accuracy of the ICD-9 codes for identifying TIA and stroke in an Italian automated database. Neurological sciences : official journal of the Italian Neurological Society and of the Italian Society of Clinical Neurophysiology. 2004;25(5):281-8.

70. Schroeck FR, Sirovich B, Seigne JD, Robertson DJ, Goodney PP. Assembling and validating data from multiple sources to study care for Veterans with bladder cancer. BMC urology. 2017;17(1):78.

71. Kim SC, Gillet VG, Feldman S, Lii H, Toh S, Brown JS et al. Validation of claims-based algorithms for identification of high-grade cervical dysplasia and cervical cancer. Pharmacoepidemiology and drug safety. 2013;22(11):1239-44.

72. Vitullo F, Di Biagio K, Murgano A, Di Bartolomeo P. Hospital discharge records as data source to monitor epidemiologic indicators of hematologic malignancies in Abruzzo. Tumori. 2016;2016(3):258-63.

73. Pennie ML, Soon SL, Risser JB, Veledar E, Culler SD, Chen SC. Melanoma outcomes for Medicare patients: association of stage and survival with detection by a dermatologist vs a nondermatologist. Arch Dermatol. 2007;143(4):488-94.

74. Omino R, Mittal S, Kramer JR, Chayanupatkul M, Richardson P, Kanwal F. The Validity of HCC Diagnosis Codes in Chronic Hepatitis B Patients in the Veterans Health Administration. Digestive diseases and sciences. 2017;62(5):1180-1185.

75. Barber EL, Doll KM, Gehrig PA. Hospital readmission after ovarian cancer surgery: Are we measuring surgical quality? Gynecologic oncology. 2017;146(2):368-372.

76. Eide MJ, Krajenta R, Johnson D, Long JJ, Jacobsen G, Asgari MM et al. Identification of patients with nonmelanoma skin cancer using health maintenance organization claims data. American journal of epidemiology. 2010;171(1):123-8.

77. Eide MJ, Tuthill JM, Krajenta RJ, Jacobsen GR, Levine M, Johnson CC. Validation of claims data algorithms to identify nonmelanoma skin cancer. The Journal of investigative dermatology. 2012;132(8):2005-9.

78. Cooper GS, Yuan Z, Stange KC, Dennis LK, Amini SB, Rimm AA. The sensitivity of Medicare claims data for case ascertainment of six common cancers. Medical care. 1999;37(5):436-44.

79. Muir KW, Gupta C, Gill P, Stein JD. Accuracy of international classification of diseases, ninth revision, clinical modification billing codes for common ophthalmic conditions. JAMA ophthalmology. 2013;131(1):119-20.

80. Gray DT, Hodge DO, Ilstrup DM, Butterfield LC, Baratz KH. Concordance of Medicare data and population-based clinical data on cataract surgery utilization in Olmsted County, Minnesota. American journal of epidemiology. 1997;145(12):1123-6.

81. Rector TS, Wickstrom SL, Shah M, Thomas Greeenlee N, Rheault P, Rogowski J et al. Specificity and sensitivity of claims-based algorithms for identifying members of Medicare+Choice health plans that have chronic medical conditions. Health services research. 2004;39(6 Pt 1):1839-57.

82. Latkany P, Duggal M, Goulet J, Paek H, Rambo M, Palmisano P et al. The need for validation of large administrative databases: Veterans Health Administration ICD-9CM coding of exudative age-related macular degeneration and ranibizumab usage. Journal of ocular biology, diseases, and informatics. 2010;3(1):30-4.

83. Keller JJ, Chung SD, Lin HC. A nationwide population-based study on the association between chronic periodontitis and erectile dysfunction. J Clin Periodontol. 2012;39(6):507-12.

84. Weiner JP, Starfield BH, Steinwachs DM, Mumford LM. Development and application of a population-oriented measure of ambulatory care case-mix. Med Care. 1991;29(5):452-72.

85. Kim HM, Smith EG, Stano CM, Ganoczy D, Zivin K, Walters H et al. Validation of key behaviourally based mental health diagnoses in administrative data: suicide attempt, alcohol abuse, illicit drug abuse and tobacco use. BMC health services research. 2012;12:18.

86. Noyes K, Liu H, Lyness JM, Friedman B. Medicare beneficiaries with depression: comparing diagnoses in claims data with the results of screening. Psychiatric services (Washington, DC). 2011;62(10):1159-66.

87. Wilchesky M, Tamblyn RM, Huang A. Validation of diagnostic codes within medical services claims. Journal of clinical epidemiology. 2004;57(2):131-41.

88. Ho TW, Ruan SY, Huang CT, Tsai YJ, Lai F, Yu CJ. Validity of ICD9-CM codes to diagnose chronic obstructive pulmonary disease from National Health Insurance claim data in Taiwan. International journal of chronic obstructive pulmonary disease. 2018;13:3055-3063.

89. Lacasse Y, Daigle JM, Martin S, Maltais F. Validity of chronic obstructive pulmonary disease diagnoses in a large administrative database. Canadian respiratory journal. 2012;19(2):e5-9.

90. Stein BD, Bautista A, Schumock GT, Lee TA, Charbeneau JT, Lauderdale DS et al. The validity of International Classification of Diseases, Ninth Revision, Clinical Modification diagnosis codes for identifying patients hospitalized for COPD exacerbations. Chest. 2012;141(1):87-93.

91. Tamariz L, Harkins T, Nair V. A systematic review of validated methods for identifying venous thromboembolism using administrative and claims data. Pharmacoepidemiology and drug safety. 2012;21 Suppl 1:154-62.

92. Semins MJ, Trock BJ, Matlaga BR. Validity of administrative coding in identifying patients with upper urinary tract calculi. The Journal of urology. 2010;184(1):190-2.

93. Nadkarni GN, Gottesman O, Linneman JG, Chase H, Berg RL, Farouk S et al. Development and validation of an electronic phenotyping algorithm for chronic kidney disease. AMIA Annual Symposium proceedings AMIA Symposium. 2014;2014:907-16.

94. Winkelmayer WC, Schneeweiss S, Mogun H, Patrick AR, Avorn J, Solomon DH. Identification of individuals with CKD from Medicare claims data: a validation study. American journal of kidney diseases : the official journal of the National Kidney Foundation. 2005;46(2):225-32.

95. Manack A, Motsko SP, Haag-Molkenteller C, Dmochowski RR, Goehring EL, Jr., Nguyen-Khoa BA et al. Epidemiology and healthcare utilization of neurogenic bladder patients in a US claims database. Neurourol Urodyn. 2011;30(3):395-401.

96. Clemens JQ, Calhoun EA, Litwin MS, McNaughton-Collins M, Kusek JW, Crowley EM et al. Validation of a modified National Institutes of Health chronic prostatitis symptom index to assess genitourinary pain in both men and women. Urology. 2009;74(5):983-7, quiz 987.e1-3.

97. Roehrborn CG. Benign prostatic hyperplasia: an overview. Rev Urol. 2005;7 Suppl 9(Suppl 9):S3-s14.

98. Souverein PC, Erkens JA, de la Rosette JJ, Leufkens HG, Herings RM. Drug treatment of benign prostatic hyperplasia and hospital admission for BPH-related surgery. Eur Urol. 2003;43(5):528-34.

99. Saigal CS, Joyce G. Economic costs of benign prostatic hyperplasia in the private sector. J Urol. 2005;173(4):1309-13.

100. Chuang S-C, Wu G-J, Lu Y-S, Lin C-H, Hsiung CA. Associations between Medical Conditions and Breast Cancer Risk in Asians: A Nationwide Population-Based Study in Taiwan. PloS one. 2015;10(11):e0143410-e0143410.

101. Fuldeore M, Yang H, Du EX, Soliman AM, Wu EQ, Winkel C. Healthcare utilization and costs in women diagnosed with endometriosis before and after diagnosis: a longitudinal analysis of claims databases. Fertil Steril. 2015;103(1):163-71.

102. Yasmeen S, Romano PS, Schembri ME, Keyzer JM, Gilbert WM. Accuracy of obstetric diagnoses and procedures in hospital discharge data. American journal of obstetrics and gynecology. 2006;194(4):992-1001.

103. Okoroh EM, Hooper WC, Atrash HK, Yusuf HR, Boulet SL. Prevalence of polycystic ovary syndrome among the privately insured, United States, 2003-2008. Am J Obstet Gynecol. 2012;207(4):299.e1-7.

104. Knaapen L, Weisz G. The biomedical standardization of premenstrual syndrome. Stud Hist Philos Biol Biomed Sci. 2008;39(1):120-34.

105. Stulberg DB, Cain LR, Dahlquist I, Lauderdale DS. Ectopic pregnancy rates in the Medicaid population. Am J Obstet Gynecol. 2013;208(4):274.e1-7.
